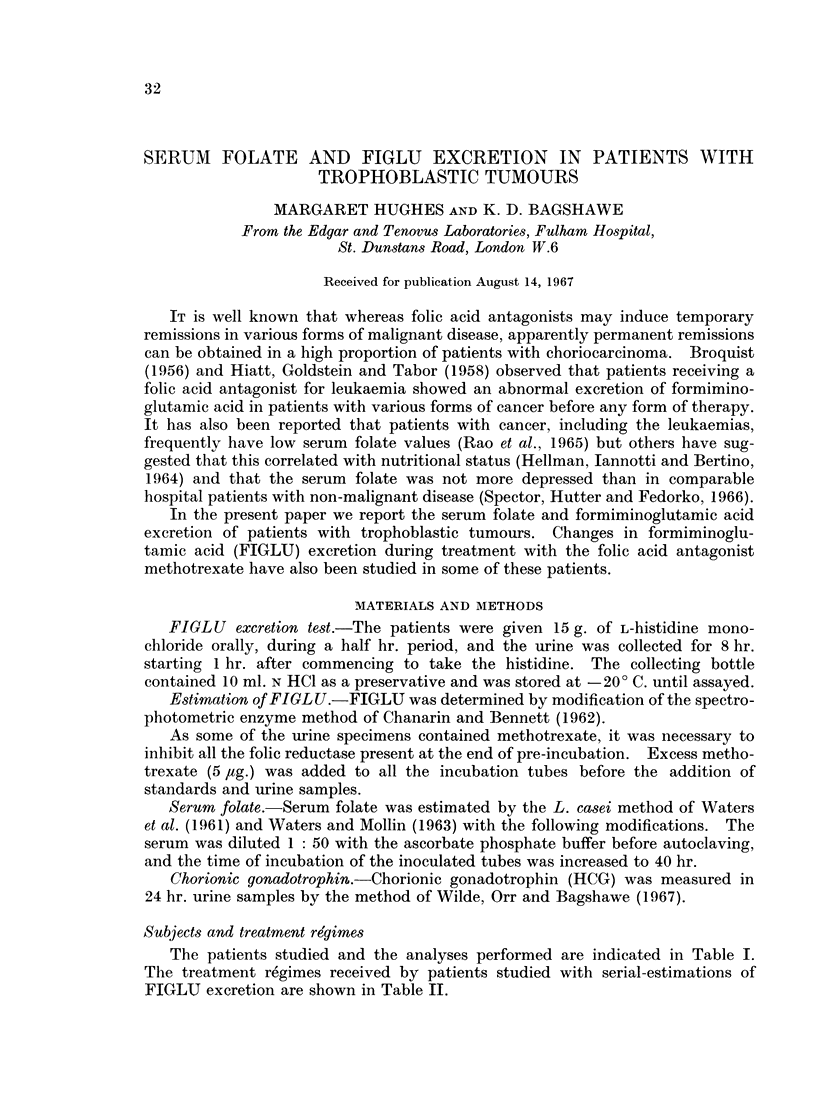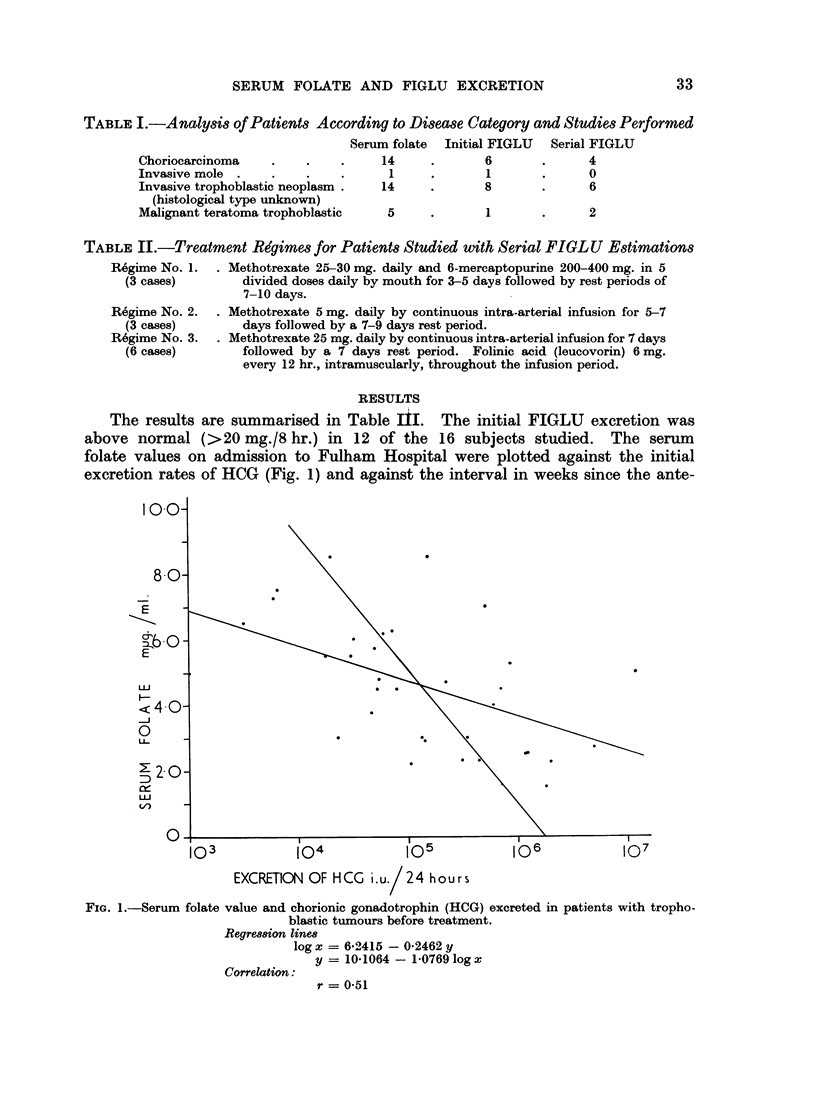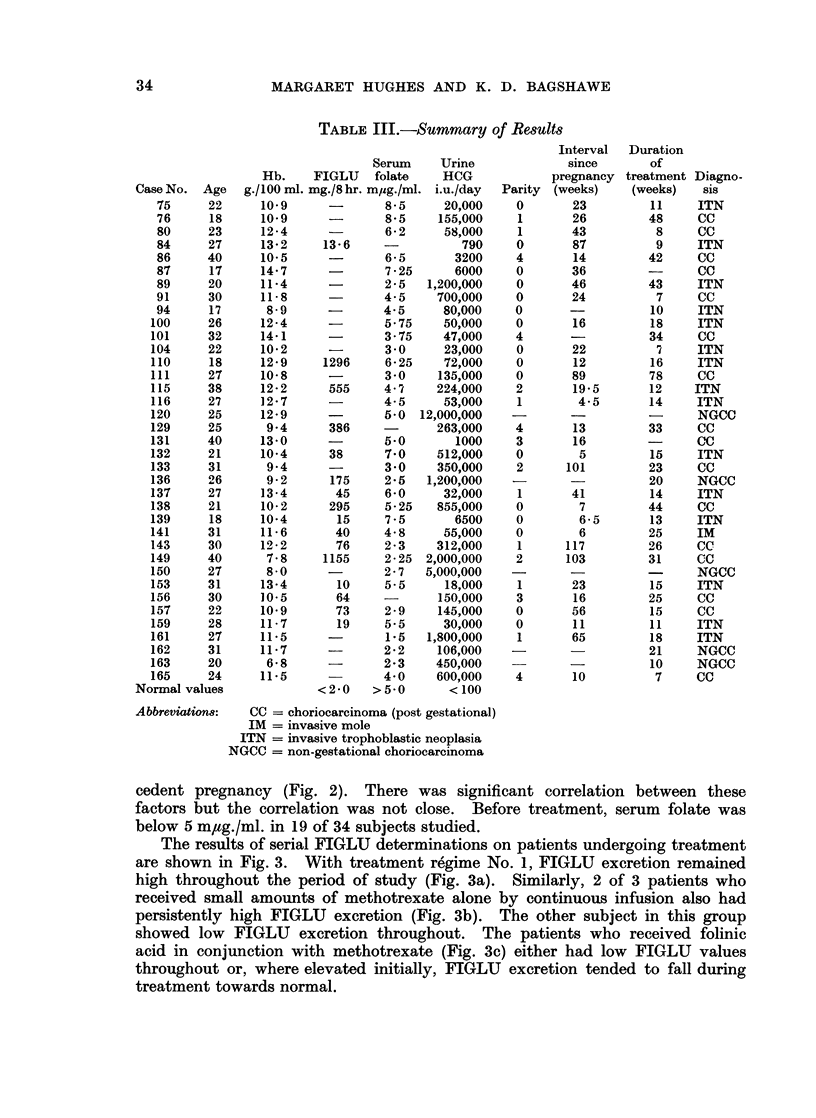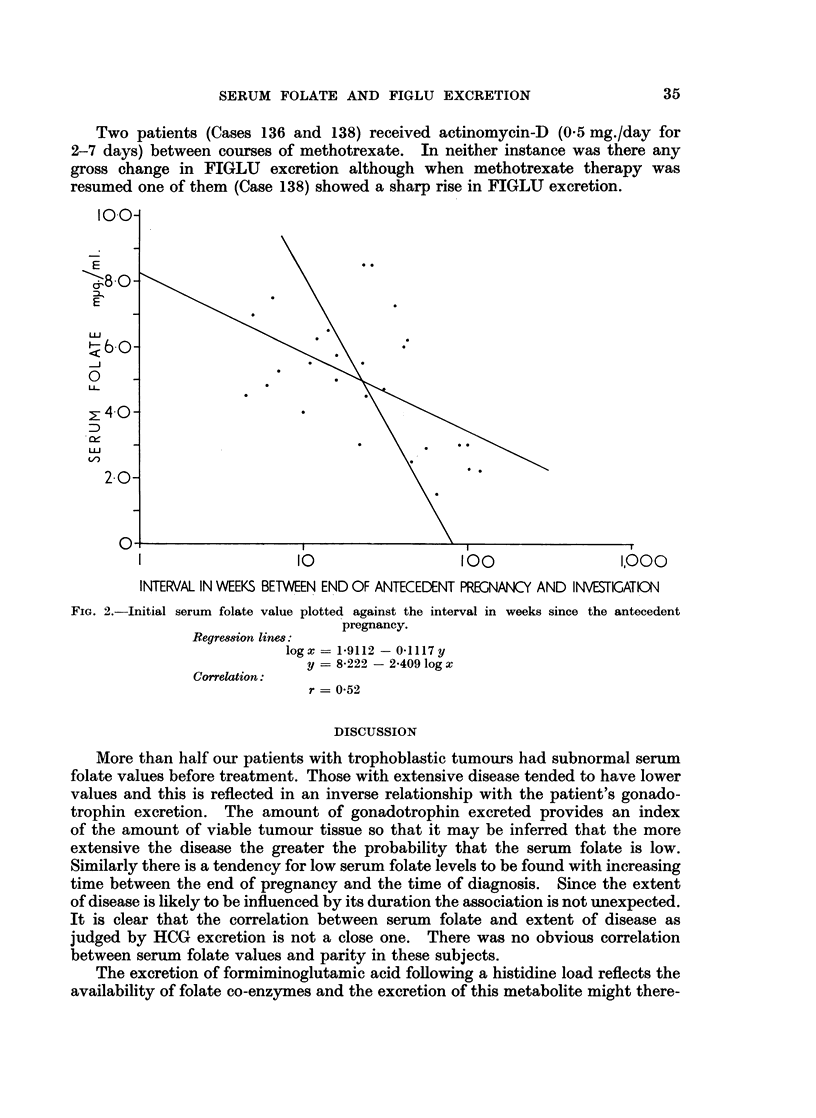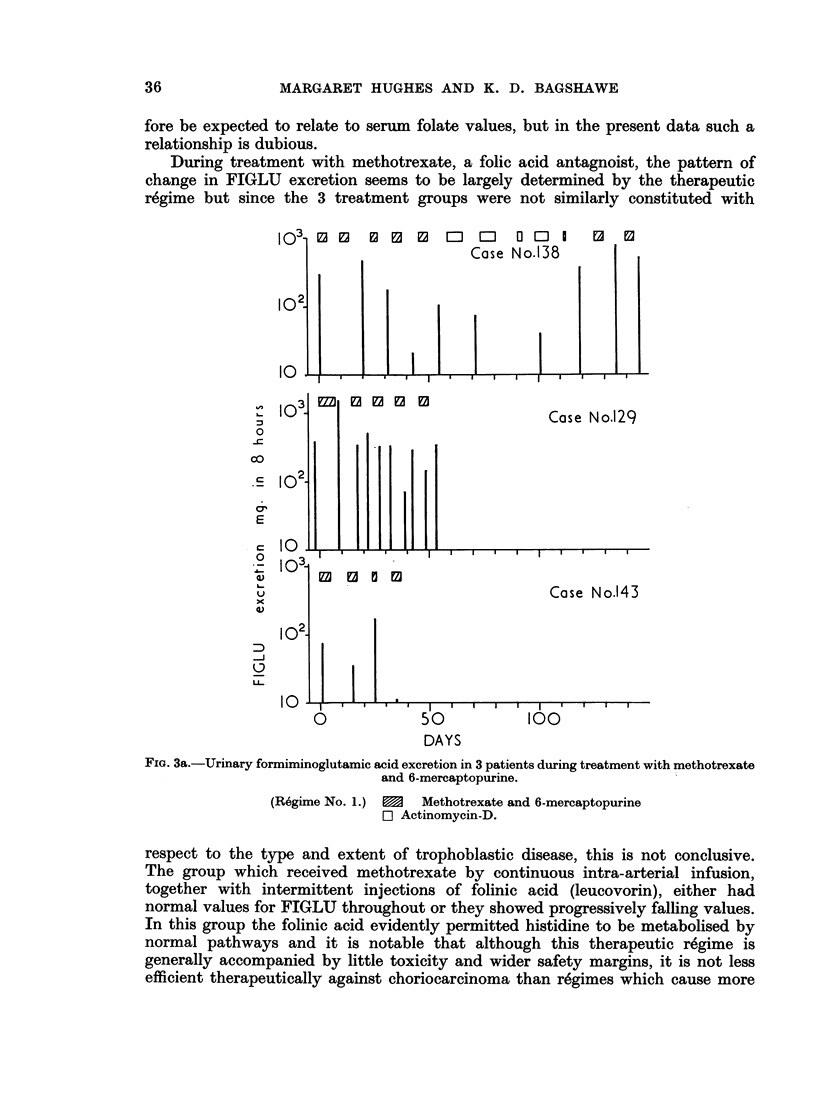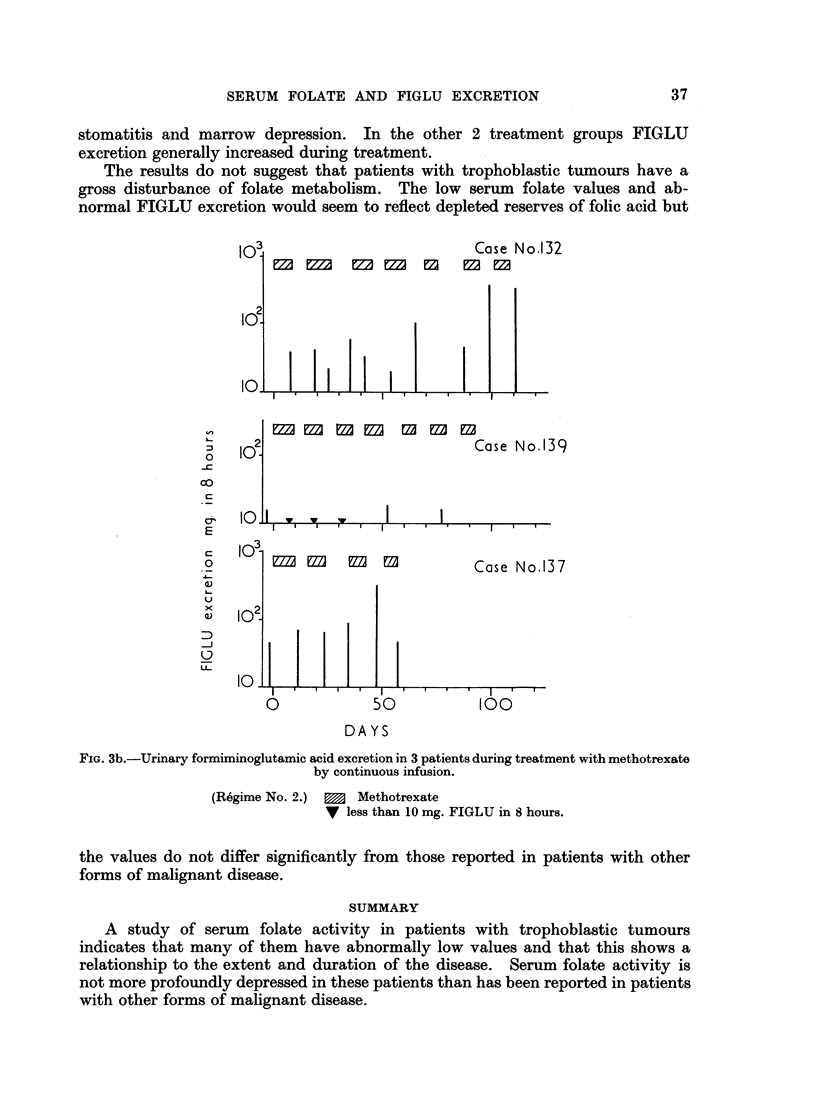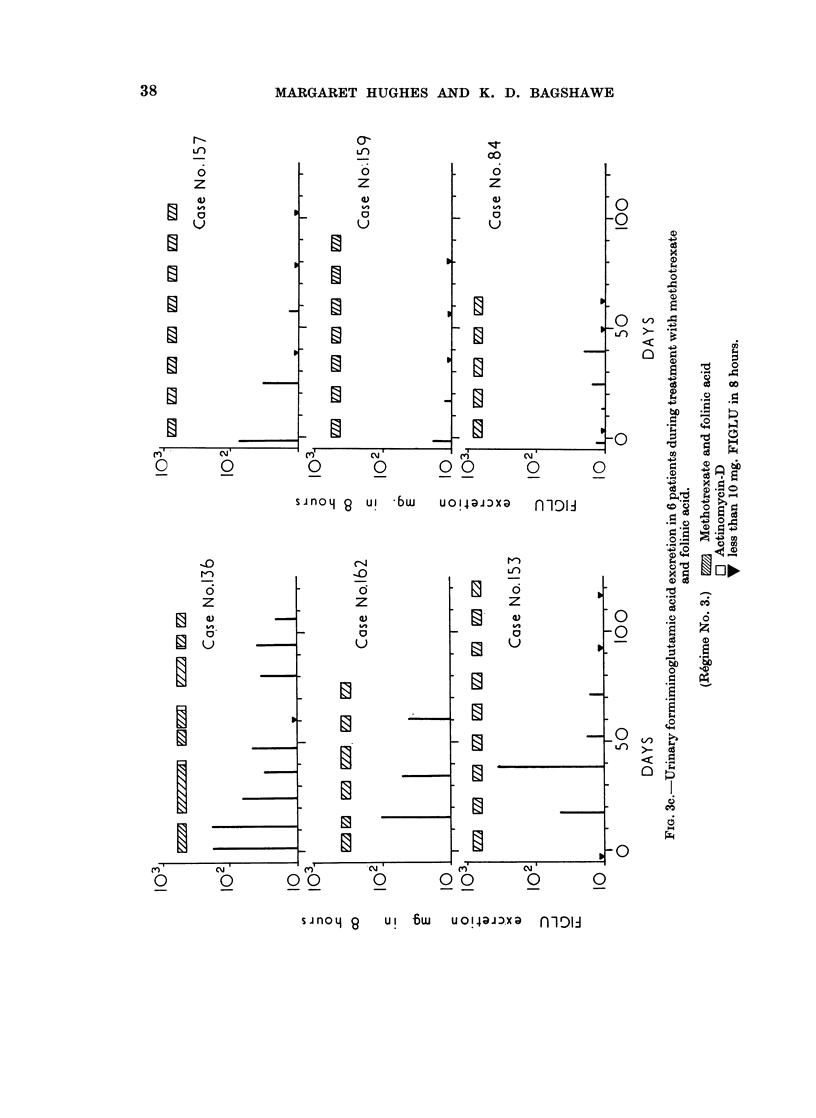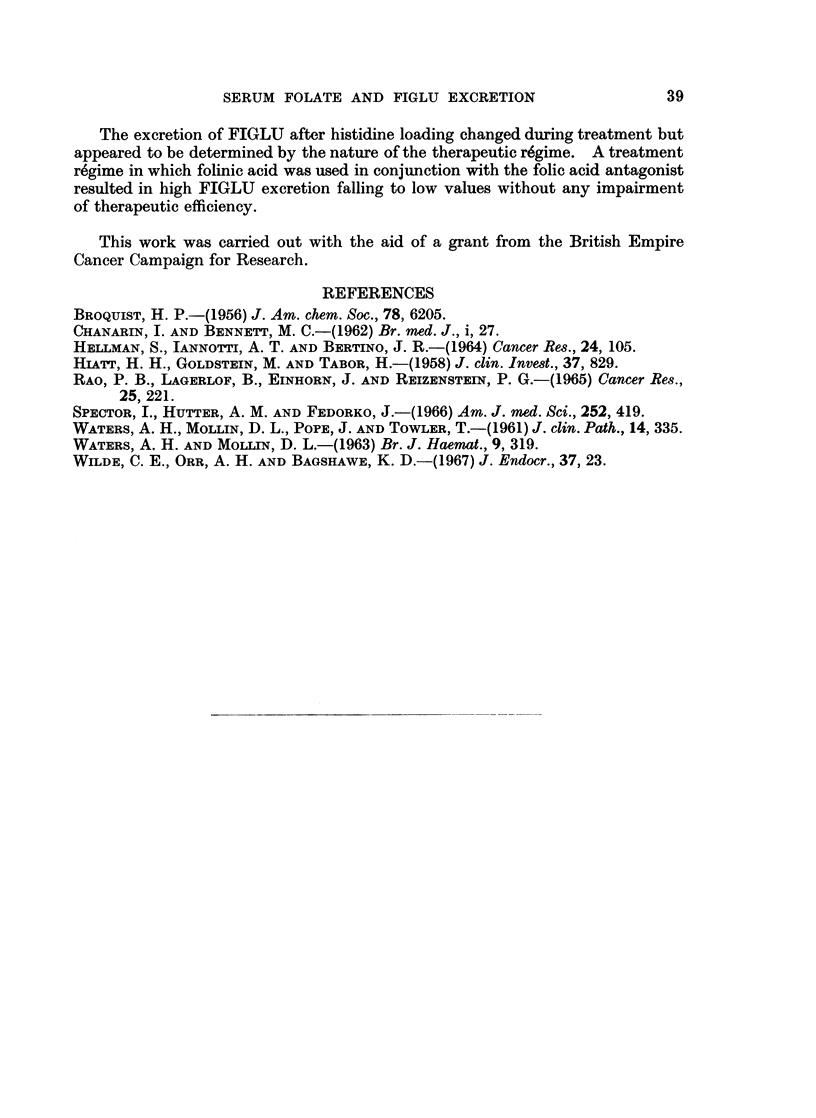# Serum folate and FIGLU excretion in patients with trophoblastic tumours.

**DOI:** 10.1038/bjc.1968.5

**Published:** 1968-03

**Authors:** M. Hughes, K. D. Bagshawe


### SERUM FOLATE AND FIGLU EXCRETION IN PATIENTS WITH TROPHOBLASTIC TUMOURS

MARGARET HUGHES AND K. D. BAGSHAWE

*From the Edgar and Tenovus Laboratories, Fulham Hospital, St. Dunstans Road, London W.6*

Received for publication August 14, 1967

It is well known that whereas folic acid antagonists may induce temporary remissions in various forms of malignant disease, apparently permanent remissions can be obtained in a high proportion of patients with choriocarcinoma. Broquist (1956) and Hiatt, Goldstein and Tabor (1958) observed that patients receiving a folic acid antagonist for leukaemia showed an abnormal excretion of formiminoglutamic acid in patients with various forms of cancer before any form of therapy. It has also been reported that patients with cancer, including the leukaemias, frequently have low serum folate values (Rao *et al.*, 1965) but others have suggested that this correlated with nutritional status (Hellman, Iannotti and Bertino, 1964) and that the serum folate was not more depressed than in comparable hospital patients with non-malignant disease (Spector, Hutter and Fedorko, 1966).

In the present paper we report the serum folate and formiminoglutamic acid excretion of patients with trophoblastic tumours. Changes in formiminoglutamic acid (FIGLU) excretion during treatment with the folic acid antagonist methotrexate have also been studied in some of these patients.

### MATERIALS AND METHODS

*FIGLU excretion test.*—The patients were given 15 g. of L-histidine monochloride orally, during a half hr. period, and the urine was collected for 8 hr. starting 1 hr. after commencing to take the histidine. The collecting bottle contained 10 ml. N HCl as a preservative and was stored at $-20°$ C. until assayed.

*Estimation of FIGLU.*—FIGLU was determined by modification of the spectrophotometric enzyme method of Chanarin and Bennett (1962).

As some of the urine specimens contained methotrexate, it was necessary to inhibit all the folic reductase present at the end of pre-incubation. Excess methotrexate (5 $\mu$g.) was added to all the incubation tubes before the addition of standards and urine samples.

*Serum folate.*—Serum folate was estimated by the *L. casei* method of Waters *et al.* (1961) and Waters and Mollin (1963) with the following modifications. The serum was diluted 1 : 50 with the ascorbate phosphate buffer before autoclaving, and the time of incubation of the inoculated tubes was increased to 40 hr.

*Chorionic gonadotrophin.*—Chorionic gonadotrophin (HCG) was measured in 24 hr. urine samples by the method of Wilde, Orr and Bagshawe (1967).

### *Subjects and treatment régimes*

The patients studied and the analyses performed are indicated in Table I. The treatment régimes received by patients studied with serial-estimations of FIGLU excretion are shown in Table II.

TABLE I.—*Analysis of Patients According to Disease Category and Studies Performed*

| | Serum folate | Initial FIGLU | Serial FIGLU |
|---|---|---|---|
| Choriocarcinoma | 14 | 6 | 4 |
| Invasive mole | 1 | 1 | 0 |
| Invasive trophoblastic neoplasm (histological type unknown) | 14 | 8 | 6 |
| Malignant teratoma trophoblastic | 5 | 1 | 2 |

TABLE II.—*Treatment Régimes for Patients Studied with Serial FIGLU Estimations*

| | |
|---|---|
| Régime No. 1. (3 cases) | Methotrexate 25–30 mg. daily and 6-mercaptopurine 200–400 mg. in 5 divided doses daily by mouth for 3–5 days followed by rest periods of 7–10 days. |
| Régime No. 2. (3 cases) | Methotrexate 5 mg. daily by continuous intra-arterial infusion for 5–7 days followed by a 7–9 days rest period. |
| Régime No. 3. (6 cases) | Methotrexate 25 mg. daily by continuous intra-arterial infusion for 7 days followed by a 7 days rest period. Folinic acid (leucovorin) 6 mg. every 12 hr., intramuscularly, throughout the infusion period. |

### RESULTS

The results are summarised in Table III. The initial FIGLU excretion was above normal (>20 mg./8 hr.) in 12 of the 16 subjects studied. The serum folate values on admission to Fulham Hospital were plotted against the initial excretion rates of HCG (Fig. 1) and against the interval in weeks since the ante-

FIG. 1.—Serum folate value and chorionic gonadotrophin (HCG) excreted in patients with trophoblastic tumours before treatment.

*Regression lines*

$$\log x = 6{\cdot}2415 - 0{\cdot}2462\, y$$

$$y = 10{\cdot}1064 - 1{\cdot}0769 \log x$$

*Correlation:*

$$r = 0{\cdot}51$$

TABLE III.—*Summary of Results*

| Case No. | Age | Hb. g./100 ml. | FIGLU mg./8 hr. | Serum folate m$\mu$g./ml. | Urine HCG i.u./day | Parity | Interval since pregnancy (weeks) | Duration of treatment (weeks) | Diagnosis |
|---|---|---|---|---|---|---|---|---|---|
| 75 | 22 | 10·9 | — | 8·5 | 20,000 | 0 | 23 | 11 | ITN |
| 76 | 18 | 10·9 | — | 8·5 | 155,000 | 1 | 26 | 48 | CC |
| 80 | 23 | 12·4 | — | 6·2 | 58,000 | 1 | 43 | 8 | CC |
| 84 | 27 | 13·2 | 13·6 | — | 790 | 0 | 87 | 9 | ITN |
| 86 | 40 | 10·5 | — | 6·5 | 3200 | 4 | 14 | 42 | CC |
| 87 | 17 | 14·7 | — | 7·25 | 6000 | 0 | 36 | — | CC |
| 89 | 20 | 11·4 | — | 2·5 | 1,200,000 | 0 | 46 | 43 | ITN |
| 91 | 30 | 11·8 | — | 4·5 | 700,000 | 0 | 24 | 7 | CC |
| 94 | 17 | 8·9 | — | 4·5 | 80,000 | 0 | — | 10 | ITN |
| 100 | 26 | 12·4 | — | 5·75 | 50,000 | 0 | 16 | 18 | ITN |
| 101 | 32 | 14·1 | — | 3·75 | 47,000 | 4 | — | 34 | CC |
| 104 | 22 | 10·2 | — | 3·0 | 23,000 | 0 | 22 | 7 | ITN |
| 110 | 18 | 12·9 | 1296 | 6·25 | 72,000 | 0 | 12 | 16 | ITN |
| 111 | 27 | 10·8 | — | 3·0 | 135,000 | 0 | 89 | 78 | CC |
| 115 | 38 | 12·2 | 555 | 4·7 | 224,000 | 2 | 19·5 | 12 | ITN |
| 116 | 27 | 12·7 | — | 4·5 | 53,000 | 1 | 4·5 | 14 | ITN |
| 120 | 25 | 12·9 | — | 5·0 | 12,000,000 | — | — | — | NGCC |
| 129 | 25 | 9·4 | 386 | — | 263,000 | 4 | 13 | 33 | CC |
| 131 | 40 | 13·0 | — | 5·0 | 1000 | 3 | 16 | — | CC |
| 132 | 21 | 10·4 | 38 | 7·0 | 512,000 | 0 | 5 | 15 | ITN |
| 133 | 31 | 9·4 | — | 3·0 | 350,000 | 2 | 101 | 23 | CC |
| 136 | 26 | 9·2 | 175 | 2·5 | 1,200,000 | — | — | 20 | NGCC |
| 137 | 27 | 13·4 | 45 | 6·0 | 32,000 | 1 | 41 | 14 | ITN |
| 138 | 21 | 10·2 | 295 | 5·25 | 855,000 | 0 | 7 | 44 | CC |
| 139 | 18 | 10·4 | 15 | 7·5 | 6500 | 0 | 6·5 | 13 | ITN |
| 141 | 31 | 11·6 | 40 | 4·8 | 55,000 | 0 | 6 | 25 | IM |
| 143 | 30 | 12·2 | 76 | 2·3 | 312,000 | 1 | 117 | 26 | CC |
| 149 | 40 | 7·8 | 1155 | 2·25 | 2,000,000 | 2 | 103 | 31 | CC |
| 150 | 27 | 8·0 | — | 2·7 | 5,000,000 | — | — | — | NGCC |
| 153 | 31 | 13·4 | 10 | 5·5 | 18,000 | 1 | 23 | 15 | ITN |
| 156 | 30 | 10·5 | 64 | — | 150,000 | 3 | 16 | 25 | CC |
| 157 | 22 | 10·9 | 73 | 2·9 | 145,000 | 0 | 56 | 15 | CC |
| 159 | 28 | 11·7 | 19 | 5·5 | 30,000 | 0 | 11 | 11 | ITN |
| 161 | 27 | 11·5 | — | 1·5 | 1,800,000 | 1 | 65 | 18 | ITN |
| 162 | 31 | 11·7 | — | 2·2 | 106,000 | — | — | 21 | NGCC |
| 163 | 20 | 6·8 | — | 2·3 | 450,000 | — | — | 10 | NGCC |
| 165 | 24 | 11·5 | — | 4·0 | 600,000 | 4 | 10 | 7 | CC |
| Normal values | | | $<2·0$ | $>5·0$ | $<100$ | | | | |

*Abbreviations*: CC = choriocarcinoma (post gestational)
IM = invasive mole
ITN = invasive trophoblastic neoplasia
NGCC = non-gestational choriocarcinoma cedent pregnancy (Fig. 2). There was significant correlation between these factors but the correlation was not close. Before treatment, serum folate was below 5 m$\mu$g./ml. in 19 of 34 subjects studied.

The results of serial FIGLU determinations on patients undergoing treatment are shown in Fig. 3. With treatment régime No. 1, FIGLU excretion remained high throughout the period of study (Fig. 3a). Similarly, 2 of 3 patients who received small amounts of methotrexate alone by continuous infusion also had persistently high FIGLU excretion (Fig. 3b). The other subject in this group showed low FIGLU excretion throughout. The patients who received folinic acid in conjunction with methotrexate (Fig. 3c) either had low FIGLU values throughout or, where elevated initially, FIGLU excretion tended to fall during treatment towards normal.

Two patients (Cases 136 and 138) received actinomycin-D (0·5 mg./day for 2–7 days) between courses of methotrexate. In neither instance was there any gross change in FIGLU excretion although when methotrexate therapy was resumed one of them (Case 138) showed a sharp rise in FIGLU excretion.

FIG. 2.—Initial serum folate value plotted against the interval in weeks since the antecedent pregnancy.

*Regression lines:*

$$\log x = 1{\cdot}9112 - 0{\cdot}1117\, y$$

$$y = 8{\cdot}222 - 2{\cdot}409 \log x$$

*Correlation:*

$$r = 0{\cdot}52$$

### DISCUSSION

More than half our patients with trophoblastic tumours had subnormal serum folate values before treatment. Those with extensive disease tended to have lower values and this is reflected in an inverse relationship with the patient's gonadotrophin excretion. The amount of gonadotrophin excreted provides an index of the amount of viable tumour tissue so that it may be inferred that the more extensive the disease the greater the probability that the serum folate is low. Similarly there is a tendency for low serum folate levels to be found with increasing time between the end of pregnancy and the time of diagnosis. Since the extent of disease is likely to be influenced by its duration the association is not unexpected. It is clear that the correlation between serum folate and extent of disease as judged by HCG excretion is not a close one. There was no obvious correlation between serum folate values and parity in these subjects.

The excretion of formiminoglutamic acid following a histidine load reflects the availability of folate co-enzymes and the excretion of this metabolite might therefore be expected to relate to serum folate values, but in the present data such a relationship is dubious.

During treatment with methotrexate, a folic acid antagnoist, the pattern of change in FIGLU excretion seems to be largely determined by the therapeutic régime but since the 3 treatment groups were not similarly constituted with

FIG. 3a.—Urinary formiminoglutamic acid excretion in 3 patients during treatment with methotrexate and 6-mercaptopurine.

(Régime No. 1.) ▨ Methotrexate and 6-mercaptopurine
□ Actinomycin-D.

respect to the type and extent of trophoblastic disease, this is not conclusive. The group which received methotrexate by continuous intra-arterial infusion, together with intermittent injections of folinic acid (leucovorin), either had normal values for FIGLU throughout or they showed progressively falling values. In this group the folinic acid evidently permitted histidine to be metabolised by normal pathways and it is notable that although this therapeutic régime is generally accompanied by little toxicity and wider safety margins, it is not less efficient therapeutically against choriocarcinoma than régimes which cause more stomatitis and marrow depression. In the other 2 treatment groups FIGLU excretion generally increased during treatment.

The results do not suggest that patients with trophoblastic tumours have a gross disturbance of folate metabolism. The low serum folate values and abnormal FIGLU excretion would seem to reflect depleted reserves of folic acid but

FIG. 3b.—Urinary formiminoglutamic acid excretion in 3 patients during treatment with methotrexate by continuous infusion.

(Régime No. 2.) ▨ Methotrexate

▼ less than 10 mg. FIGLU in 8 hours.

the values do not differ significantly from those reported in patients with other forms of malignant disease.

### SUMMARY

A study of serum folate activity in patients with trophoblastic tumours indicates that many of them have abnormally low values and that this shows a relationship to the extent and duration of the disease. Serum folate activity is not more profoundly depressed in these patients than has been reported in patients with other forms of malignant disease.

FIG. 3c.—Urinary formiminoglutamic acid excretion in 6 patients during treatment with methotrexate and folinic acid.

(Régime No. 3.) ▨ Methotrexate and folinic acid

□ Actinomycin-D

▼ less than 10 mg. FIGLU in 8 hours.

The excretion of FIGLU after histidine loading changed during treatment but appeared to be determined by the nature of the therapeutic régime. A treatment régime in which folinic acid was used in conjunction with the folic acid antagonist resulted in high FIGLU excretion falling to low values without any impairment of therapeutic efficiency.

This work was carried out with the aid of a grant from the British Empire Cancer Campaign for Research.

### REFERENCES

Broquist, H. P.—(1956) *J. Am. chem. Soc.*, **78**, 6205.
Chanarin, I. and Bennett, M. C.—(1962) *Br. med. J.*, i, 27.
Hellman, S., Iannotti, A. T. and Bertino, J. R.—(1964) *Cancer Res.*, **24**, 105.
Hiatt, H. H., Goldstein, M. and Tabor, H.—(1958) *J. clin. Invest.*, **37**, 829.
Rao, P. B., Lagerlof, B., Einhorn, J. and Reizenstein, P. G.—(1965) *Cancer Res.*, **25**, 221.
Spector, I., Hutter, A. M. and Fedorko, J.—(1966) *Am. J. med. Sci.*, **252**, 419.
Waters, A. H., Mollin, D. L., Pope, J. and Towler, T.—(1961) *J. clin. Path.*, **14**, 335.
Waters, A. H. and Mollin, D. L.—(1963) *Br. J. Haemat.*, **9**, 319.
Wilde, C. E., Orr, A. H. and Bagshawe, K. D.—(1967) *J. Endocr.*, **37**, 23.